# Authors’ Classification of Sphenoid Sinus Pneumatizations into the Sphenoid Bone Processes

**DOI:** 10.3390/jcm14165811

**Published:** 2025-08-17

**Authors:** Przemysław Kiciński, Michał Podgórski, Piotr Grzelak, Beata Małachowska, Michał Polguj

**Affiliations:** 1 Department of Angiology, Chair of Anatomy and Histology, Medical University of Lodz, 90-419 Lodz, Poland; 2 III Department of Radiology and Diagnostic Imaging, Medical University of Lodz, 90-419 Lodz, Poland; michal.podgorski@umed.lodz.pl; 3 Department of Diagnostic Imaging, Polish Mother’s Memorial Hospital Research Institute, 93-338 Lodz, Poland; piotr.grzelak@iczmp.edu.pl; 4 Department of Radiation Oncology, Albert Einstein College of Medicine, Bronx, NY 10461, USA; beata.malachowska@einsteinmed.org; 5 Department of Normal and Clinical Anatomy, Chair of Anatomy and Histology, Medical University of Lodz, 90-419 Lodz, Poland; michal.polguj@umed.lodz.pl

**Keywords:** sphenoid sinus, sphenoid bone processes, classification, paranasal sinus

## Abstract

**Background**: The varied shape and size of the sphenoid sinuses result in a highly variable degree of extension, described in different ways in the literature. The aim of the study was to create the authors’ classification of the sphenoid sinus extensions into the sphenoid bone processes. **Methods**: The study was retrospective, based on the results of head CT scans. The study group comprised 432 children, aged from birth to 18 years. Three types of sphenoid sinus extension into the sphenoid bone processes were proposed. Pneumatization of the lesser wing (type I), of the greater wing (type II), and of the pterygoid process (type III). Three subtypes were defined for each type. Pneumatization of the lesser wing in relation to the optic canal: only above (Ia), only below (Ib), and simultaneously above and below (Ic). Pneumatization of the greater wing in relation to the foramen rotundum: only above (IIa), only below (IIb), and simultaneously above and below (IIc). Pneumatization of the pterygoid process in relation to the pterygoid canal: only laterally (IIIa), only medially (IIIb), and simultaneously laterally and medially (IIIc). **Results**: Pneumatization of the lesser wings was observed in 19.44%, of the greater wings in 36.11%, and of the pterygoid processes in 25.00 of % children. As a result of the analysis conducted in accordance with the proposed classification, bilateral pneumatization of the lesser wings (type I), greater wings (type II), and pterygoid processes (type III) were found to occur more frequently than unilateral pneumatization. In the case of rare subtypes (Ia, Ic, IIa, Iic, or IIIb), a given subtype was observed to occur more frequently unilaterally. **Conclusions**: In the conducted analysis, we presented the authors’ classification of sphenoid sinus extensions into the sphenoid bone processes.

## 1. Introduction

The sphenoid bone consists of an unpaired body of the sphenoid bone and paired processes: the lesser wings, the greater wings, and the pterygoid processes of the sphenoid bone. In the sphenoid bone, two pneumatic spaces develop, divided by a septum, called the sphenoid sinuses, which first pneumatize the body of the sphenoid bone and in the following years of life may also extend to the lesser and/or greater wings and/or the pterygoid processes of the sphenoid bone [1,2,3,4,5].

In the literature, the extensions of the sphenoid sinuses are described by the authors in various ways, among others, as recesses or in classifications as defined types and subtypes. Furthermore, pneumatization of the sphenoid bone leads to exposure of certain anatomical structures in the sphenoid sinus. Examples of recesses that are the sphenoid sinus extensions into the processes of the sphenoid bone are optico-carotid, lateral, posterolateral, anterolateral, and pterygoid recess [2,6,7]. As a result of the pneumatization of sphenoid bone processes, important anatomical structures such as the optic nerve (II), the maxillary nerve (V2), the nerve, artery, and vein of the pterygoid canal, or the internal carotid artery may be exposed in the sphenoid sinus [8,9,10,11]. The exposure of specific anatomical structures is associated with the development of the sphenoid sinuses [7,12]. The known classifications of the sphenoid sinuses do not include all sphenoid sinus extensions into the sphenoid bone processes [13,14,15,16]. Individually, sphenoid sinuses are highly variable in shape and size and can extend in different directions. High variability of sphenoid sinus extensions does not facilitate the creation of a simple classification of the sphenoid sinuses. At the same time, knowledge of the sphenoid bone and sphenoid sinus structure due to their location and numerous anatomical relationships plays an important role in imaging studies and beyond. This knowledge is essential and important when planning surgery within the skull or in sphenoid endoscopic surgery. The degree of the sphenoid bone pneumatization is important in the spread of pathology from the sphenoid sinuses to adjacent anatomical structures and vice versa [3,5,12,13,17,18].

The aim of the study was to create the authors’ classification of the sphenoid sinus extensions into the sphenoid bone processes: the lesser wing, the greater wing, and the pterygoid process in the frontal plane in relation to the optic canal, the foramen rotundum and the pterygoid (Vidian) canal, respectively, in children on the basis of computed tomography imaging.

## 2. Materials and Methods

This was a retrospective study, conducted at the Department of Diagnostic Imaging, Polish Mother’s Memorial Hospital–Research Institute. It comprised 432 children, 216 girls and 216 boys (in each year of life, the number of girls and boys was equal), aged from birth to 18 years, who underwent head CT and met the inclusion criteria. All examinations were performed with a 256-row Philips Brilliance computed CAT (computerized axial tomography) scanner. All children’s parents and/or legal guardians gave informed consent for head computed tomography investigation and its use for scientific research. The inclusion criterion was a correctly performed head CT. The exclusion criteria were the following: disease of the paranasal sinuses or pathology within them, traumatic cranial bone and head changes, condition after head surgery, cranial deformation, genetic or metabolic disease, congenital or acquired developmental defect, history of growth disorders, active neoplastic process, artifacts that make impossible the skull, and paranasal sinuses assessment [19].

The evaluation of the sphenoid sinus extensions into the sphenoid bone processes was performed in relation to the optic canal, the foramen rotundum, and the pterygoid canal. Each of the lesser wings runs laterally and begins with two roots—an anterior root and a posterior root—which encompass the optic canal. Therefore, the optic canal is located at the junction of the lesser wing with the body of the sphenoid bone. The greater wings of the sphenoid bone are attached to the lateral surfaces of the sphenoid bone by a narrowed part and are directed laterally. Above the attachment of each greater wing, there is a groove for the vertebral artery. The foramen rotundum is situated slightly lateral to the base of the greater wing and is very well visualized using imaging technology. Therefore, the boundary between the body and the greater wing was assumed to be the sagittal plane running through the medial edge of the foramen magnum. The pterygoid processes of the sphenoid bone extend bilaterally downwards at the junction of the body and the greater wing. The base of the pterygoid process is pierced sagittally by the pterygoid canal. Therefore, the boundary between the body and the pterygoid process was assumed to be the transverse plane running through the pterygoid canal [1,4,13,14,15,20,21].

**Figure 1:**
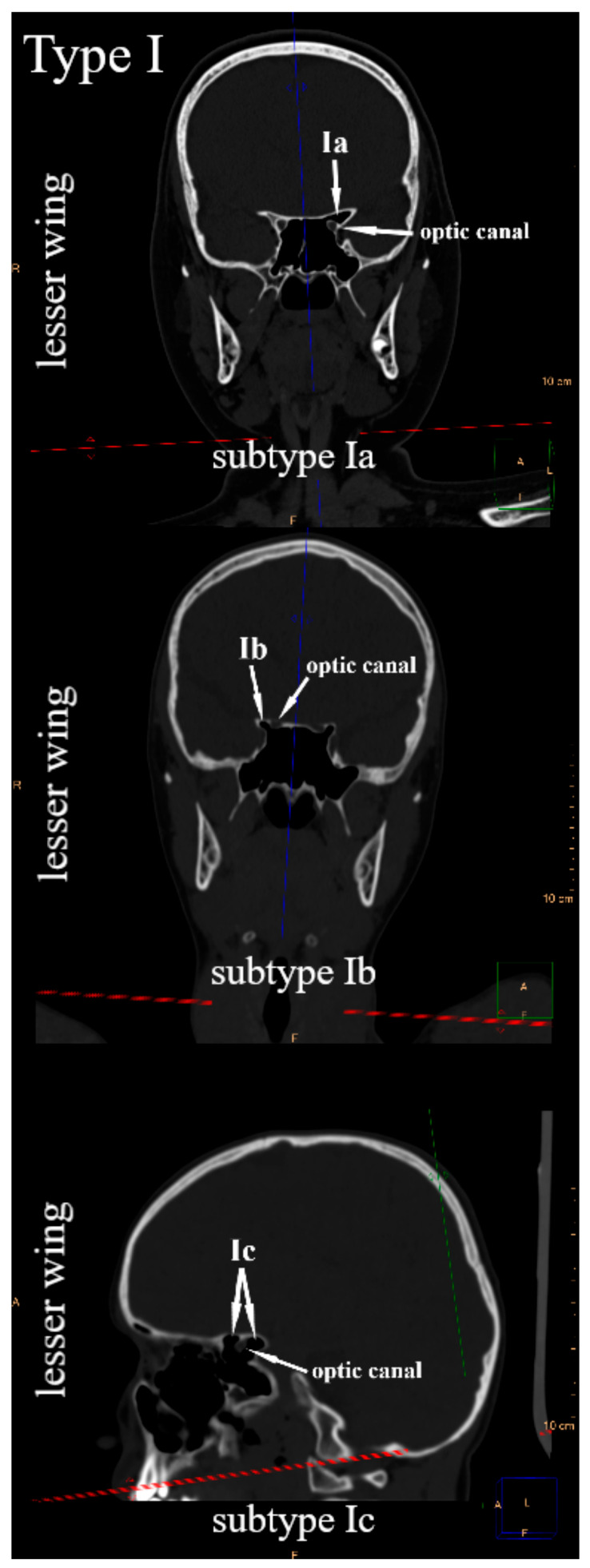
Type I and subtypes of type I—sphenoid sinus extensions into the lesser wing.

**Figure 2:**
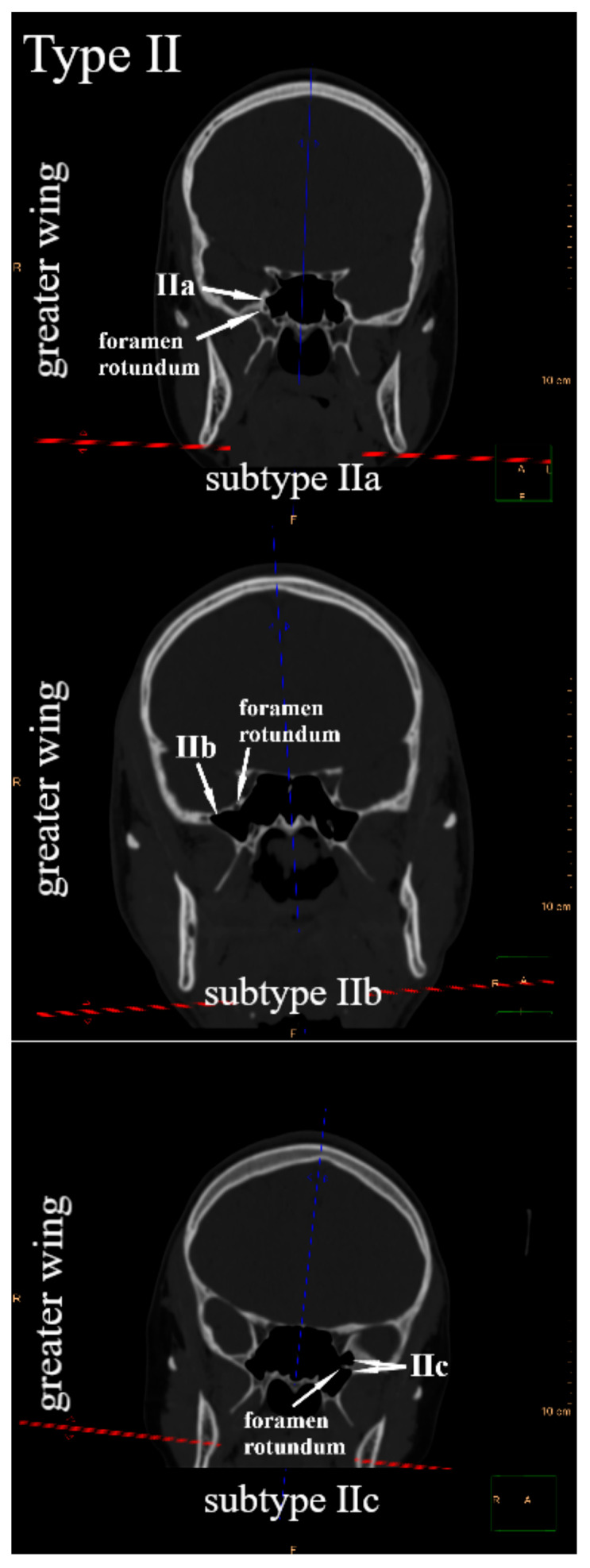
Type II and subtypes of type II—sphenoid sinus extensions into the greater wing.

**Figure 3:**
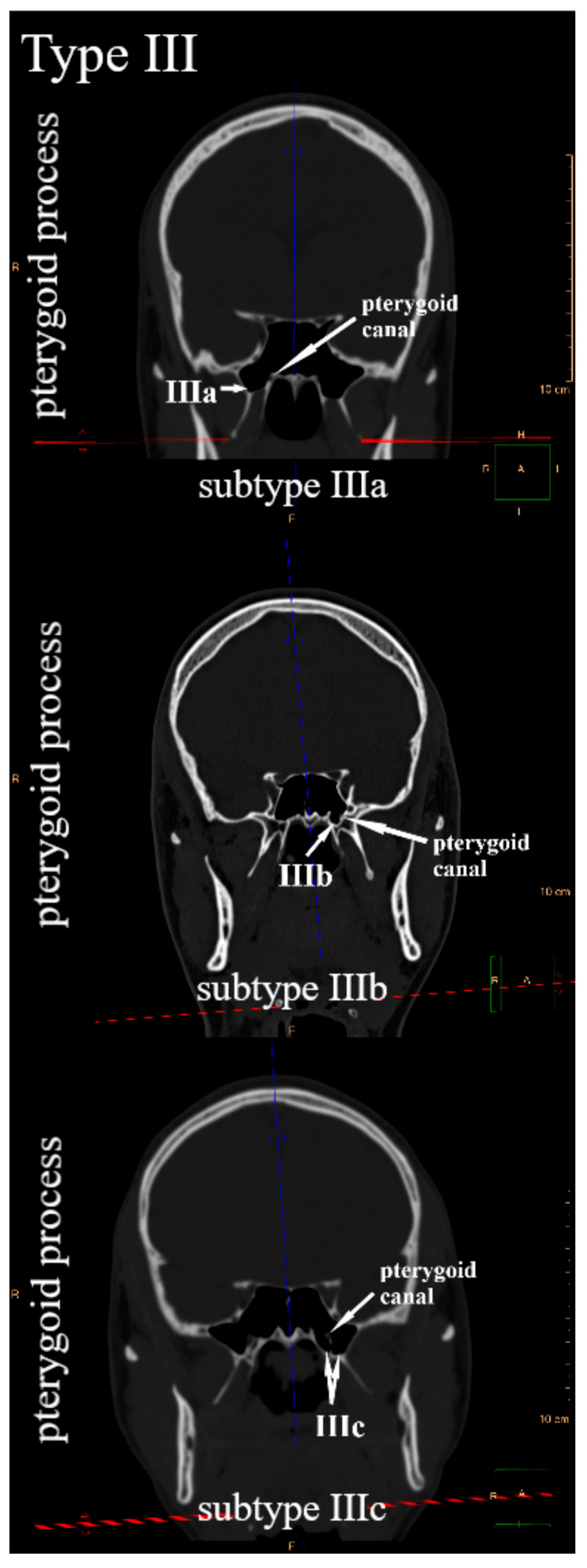
Type III and subtypes of type III—sphenoid sinus extensions into the pterygoid process.

Due to the position of the lesser wings, the greater wings, and the pterygoid processes in relation to the body of the sphenoid bone, the frontal plane was suggested to systematize their pneumatization. Since the sphenoid bone has three pairs of sphenoid processes, three types of sphenoid sinus extensions were distinguished. The sphenoid sinus extends into the lesser wing—type I, the greater wing—type II, and the pterygoid process—type III. Within each type, three subtypes were defined, respectively. It was observed that in the frontal plane, the sphenoid sinus in relation to the optic canal may extend into the lesser wing: only above—subtype Ia (anterior root of the lesser wing), only below—subtype Ib (posterior root of the lesser wing), or simultaneously above and below—subtype Ic (Figure 1). The sphenoid sinus can pneumatize the greater wing in relation to the foramen rotundum: only above—subtype IIa, only below—subtype IIb, or simultaneously above and below—subtype IIc (Figure 2). In relation to the pterygoid canal, it may extend into the pterygoid process, only laterally—subtype IIIa, only medially—subtype IIIb, or simultaneously laterally and medially—subtype IIIc (Figure 3). All types and subtypes are presented in Table 1.

Statistical analysis. Descriptive statistics values for continuous variables are presented as mean and standard deviation, and 95% CI (confidence interval). Student’s *t*-test was used to compare a continuous variable between two groups. Nominal variables were given as numbers along with the percentage from the corresponding group. McNemar’s test and Fisher’s Exact test were used to compare nominal variables between groups. STATISTICA 13.1 (TIBCO Software, Palo Alto, CA, USA) was used to perform statistical analysis. *p* < 0.05 was considered statistically significant.

## 3. Results

The mean age of the study group was 9.01 ± 5.20 years (95% Cl, 8.52–9.51); girls: 9.03 ± 5.21 years (95% Cl, 8.33–9.73); and boys: 9.00 ± 5.20 years (95% Cl, 8.30–9.69); *p* = 0.9426. In the study group, the presence of the right and left sphenoid sinus was detected in 427 (98.84%) children (214 girls and 213 boys), whereas in 5 (1.16%) patients, they did not develop in the first year of life (2 girls and 3 boys). Sphenoid sinus with lesser wings pneumatization was observed in 84 (19.44%), with greater wings in 156 (36.11%), and with pterygoid processes in 108 (25.00%) children.

In accordance with the classification proposed above, Table 2 presents the frequency of occurrence of individual types and subtypes: in the right sphenoid sinus, in the left sphenoid sinus, in the right and left sphenoid sinus simultaneously, and lack of pneumatization of the sphenoid bone processes. For all types and subtypes, the highest percentage constituted children who have not yet had sphenoid bone processes of pneumatization. The high percentage of children without pneumatization of the sphenoid bone processes is due to the fact that the study group consists of children from birth to 18 years of age whose sphenoid sinuses are still in the process of development. In the investigated group of children, for each type, a statistically significantly more frequent occurrence of bilateral pneumatization of the lesser wings (type I), greater wings (type II), and pterygoid processes (type III) was found than unilateral pneumatization. Pneumatization of individual processes of the sphenoid bone develops more often symmetrically than asymmetrically. However, when analyzing the subtypes, it was observed that in the case of rare extensions of the sphenoid sinuses into the sphenoid bone processes (subtypes: Ia, Ic, IIa, IIc or IIIb), a unilateral occurrence of a given subtype was observed more often (only in the right or only in the left sphenoid sinus in a given child) (Table 2).

Analyzing the frequency of individual types and subtypes depending on gender, a statistically significantly more frequent pneumatization of the greater wings (type II) was found in girls than in boys (*p* = 0.0074) in the right sphenoid sinus. At the same time, subtype IIb in the right sphenoid sinus was also statistically significantly more frequent in girls than in boys. The frequency of occurrence of individual types and subtypes in the right and left sphenoid sinuses for girls and boys, respectively, is presented in Table 3.

## 4. Discussion

In the conducted study, we suggested the authors’ classification of the sphenoid sinus extension in the frontal plane into individual sphenoid bone processes. Three types of extensions were defined in the classification. Then, three subtypes were defined for each type considering the way the sphenoid sinus extended into individual sphenoid bone processes with respect to the optic canal, the foramen rotundum, and the pterygoid canal. The classification allows for the organization of sphenoid sinus pneumatization patterns into the sphenoid bone processes.

One of the better-known classifications of the sphenoid bone pneumatization is that proposed by Hammer and Radberg [2,22]. It concerns the level of pneumatization of the sphenoid bone body. Depending on the variant of pneumatization of the sphenoid bone body in relation to the sella turcica, on the sagittal plane, in the anteroposterior direction, the authors distinguished its three groups: conchal, presellar, and sellar type [2,23]. Modifications to this classification consist of distinguishing the fourth type, in which the sphenoid sinus extends beyond the posterior wall of the sella turcica and is referred to as the postsellar type [3,16,17] or as the complete sellar type, defining the third type as the incomplete sellar type [21]. Further sphenoid sinus pneumatization beyond the sphenoid bone body into the clivus on the sagittal plane can occur: over the floor of the sella turcica or towards the basilar part of the occipital bone below the plane defined by the pterygoid canals or in both directions simultaneously [2,21]. Wang et al. proposed a classification in which they distinguished: sphenoid body type—the sphenoid sinus pneumatizes only the sphenoid body, clival type—including extensions of the sphenoid sinus on the sagittal plane, anterior type, i.e., anterior recess—extension towards the maxillary sinus, combined type, including more than one type in the same sphenoid sinus. They also distinguished as individual types—the lesser wing type involving pneumatization of the lesser wing by the lesser wing posterior root into the anterior clinoid process. The last type is the lateral type, which includes greater wing-type—pneumatization of the greater wing below the foramen rotundum; pterygoid type—pneumatization of the pterygoid process lateral to the pterygoid canal; and full lateral type—the sphenoid sinus passes below the line connecting the foramen rotundum to the pterygoid canal, defined as the Vidian to Rotundum line (VR line), towards the greater wing and the pterygoid process [13]. In the classification of Bilgir et al., pneumatization of the sphenoid bone was classified in two directions: posteroanterior and lateral, distinguishing types and subtypes in both directions. Four types of lateral pneumatization of the sphenoid bone were distinguished: lateral body, lesserwing, inferior, and combined (lesserwing–inferior). The lesserwing type pneumatization went from the optic canal to the anterior clinoid process. In the inferior type, four subtypes were distinguished, involving extensions of the sphenoid sinus below the VR line. Inferior body subtype—extension of the sphenoid sinus below the VR line, without orientation as to whether it is the greater wing or the pterygoid process; pterygoid inferior subtype—into the pterygoid process lateral to the pterygoid canal; greaterwing inferior subtype—into the greater wing below the foramen rotundum; full lateral subtype—simultaneously into the greater wing and the pterygoid process between the foramen rotundum and the pterygoid canal [15]. Dal Secchi et al. distinguished and illustrated four types in the lateral extension in the frontal plane: lesser wing type—pneumatization of the lesser wing only below the optic canal; greater wing type—pneumatization of greater wing extends laterally to the foramen rotundum; pterygoid type—pneumatization of the pterygoid process lateral to the pterygoid canal; and complete type—lateral extension simultaneously into the greater wing and the pterygoid process between the foramen rotundum and the pterygoid canal [4]. Jaworek-Troć et al. distinguished in lateral pneumatization: the anterolateral recess—in the direction of the lesser wing over the optic canal; the posterolateral recess—in the direction of the lesser wing below the optic canal; the lateral recess—in the direction of the greater wing and the pterygoid process beyond the VR line; the pterygoid recess—in the direction of the pterygoid process lateral to the pterygoid canal; and the palatine recess—in the direction of the palatine bone [6].

The known classifications that define the so-called lateral extensions or pneumatizations of the sphenoid sinus are based on the assessment in relation to the VR line or of the lateral recess of the sphenoid sinus, which extends between the foramen rotundum and the pterygoid (Vidian) canal [4,14,16]. When assessing the pneumatization of the greater wing and the pterygoid process in relation to the VR line, extensions of the sphenoid sinus above the foramen rotundum into the greater wing and medially from the pterygoid canal into the pterygoid process are not included. The sphenoid sinus extension into the greater wing may extend not only below, but also above or bilaterally in relation to the foramen rotundum. This is illustrated by studies assessing the degree of exposure or protrusion of the maxillary nerve into the sphenoid sinus [3,7,8,9,10]. Our study confirms the possibility of pneumatization of the greater wing above the foramen rotundum, and, in our classification, it was defined as subtype IIa. However, the pneumatization of the greater wing both above and below the foramen rotundum is subtype IIc. In the case of pterygoid processes, cases of their pneumatization were described not only lateral to the pterygoid canal but also medially [2,7]. This is confirmed by studies assessing the exposure of the pterygoid nerve and vessels in the sphenoid sinus [8,9,11,24]. If pneumatization of the sphenoid sinus into the pterygoid process occurs simultaneously laterally and medially and affects both the right and left sinuses, the pterygoid nerves and vessels protrude into the sinus lumen and, due to their appearance, are referred to as “crab eye” [2]. The possibility of sphenoidal sinus pneumatization into the pterygoid process medially, laterally, and bilaterally from the pterygoid canal has also been confirmed by our study. Pneumatization of the sphenoid sinus into the lesser wing can occur via both the anterior and posterior roots. The sphenoid sinus extension into the anterior root, above the optic nerve, is referred to as the anterolateral recess [6]. The sphenoid sinus extension below the optic nerve and over the internal carotid artery into the posterior root of the lesser wing is defined as the optic-carotid recess. Its depth may vary, and it extends towards the anterior clinoid process of the sphenoidal bone [2]. It can be found in the literature that pneumatization of the anterior clinoid process forms the optico-carotid recess [3]. The pneumatization of the sphenoid sinus in the direction of the optic nerve into the posterior root is sometimes described as a posterolateral recess [6]. Andrianakis et al. proposed a classification of optic-carotid recess and anterior clinoid process pneumatization. The authors defined the optic-carotid recess as a depression between the optic nerve and the internal carotid artery. Depending on the extension of the optic-carotid recess in relation to the position of the optic nerve, they distinguished two subtypes: sub-optical and latero-optical optic-carotid recess. They defined the recess above the optic nerve as the supra-optic recess. However, the presence of both latero-optical optic-carotid recess and supra-optic recess on the same side was defined as peri-optical recess [25]. Our classification has confirmed that the sphenoid sinus can extend into the lesser wing via the anterior root, i.e., above the optic nerve, or into the posterior root, i.e., below the optic nerve, and it can also extend simultaneously on the same side above and below the optic nerve. Furthermore, our classification allows us to systematize the possibility of pneumatization of all sphenoid processes in relation to the optic canal, the foramen rotundum, and the pterygoid canal.

The importance of pneumatization of the sphenoid bone as a clinical problem is demonstrated by numerous studies, which also illustrate the high percentage of adults who experience pneumatization of the sphenoid bone processes [4,6,7,13,15,20,21]. Since our study concerns children in whom the process of pneumatization of the sphenoid bone progresses in the subsequent years of life, the obtained data cannot be easily compared with those obtained in studies on adults [26,27]. Available research shows that the volume of the sphenoid sinuses plays a role in the occurrence of specific types, dilatations, or recesses [12,28]. Due to different methodological approaches and nomenclature used in individual studies, there are often significant differences in the frequency of pneumatization of individual sphenoid bone processes. Wang et al. observed pneumatization of the greater wings in 12.0% and of the pterygoid processes in 10.9%, whereas the full lateral type was found in 77.1% of the study participants [13]. Famurewa et al. found pneumatization of the lesser wings in 11.6%, of the greater wings in 35.0%, of the pterygoid processes in 45.1%, and the full lateral type in 30.3% of the study group [20]. Dal Secchi et al. revealed pneumatization of the lesser wings in 13%, of the greater wings in 47%, of the pterygoid processes in 1%, and of the complete type in 23% of the study group [4]. Jaworek-Troć et al. estimated the incidence of posterolateral recess, i.e., pneumatization below the optic canal, at 32.09%, and of anterolateral recess, i.e., above the optic canal, at 27.36%. The incidence of the lateral recess was estimated at 65.88% and that of the pterygoid recess at 42.57% [6]. In these studies, the assessment of the pneumatization of the greater wings, pterygoid processes, and the full lateral type was made with reference to the VR line, i.e., below the foramen rotundum and lateral to the pterygoid canal. In our classification, we did not propose a type in relation to the VR line. According to Bilgir et al., a slight protrusion beyond the VR line does not define whether we have pneumatization of the greater wing or the pterygoid process [15]. Applying our classification, simultaneous extension of the sphenoid sinus into the greater wing and the pterygoid process between the foramen rotundum and the pterygoid canal can be described by combining appropriate subtypes, e.g., IIb or IIc with IIIa or IIIc. By providing subtypes, we also inform whether the extension is unilateral or bilateral in relation to the anatomical structures going through the foramen rotundum or the pterygoid canal, which is of clinical importance [8,17,24]. Moreover, listing the subtypes allows us to recognize the type of sphenoid sinus extensions into the sphenoid bone processes found there.

Due to clinical importance, numerous studies also evaluate the protrusion of specific anatomical structures into the sphenoid sinus in adults [8,9,11,17,18]. For example, Unal et al. detected the protrusion of the optic nerve into the sphenoid sinus in 31.3%, of the maxillary nerve in 30.3%, and of the pterygoid nerve in 35.7% of the examined cases [8]. However, Hewaidi et al. reported the optic nerve protrusion into the sphenoid sinus in 35.7%, the maxillary nerve in 24.3% and the pterygoid nerve in 27% of cases [9]. In our classification, subtypes Ic, IIc, and IIIc define bilateral pneumatization into the optic canal, foramen rotundum, and pterygoid canal, i.e., into the optic nerve, maxillary nerve, and pterygoid nerve and vessels, respectively.

The limitations of the study are that the frequency of individual types and subtypes applies to children whose sphenoid sinuses are in the process of development. Thus, the percentage of each type and subtype will be different in adults and may differ between geographic locations. Therefore, it is worth considering conducting a similar analysis in a group of adults and a different location with respect to the proposed types and subtypes, taking into account all possibilities of the sphenoid sinus extension into the sphenoid bone processes.

## 5. Conclusions

The conducted analysis presents the authors’ classification of sphenoid sinus extensions into the sphenoid bone processes: the greater wing, the lesser wing, and the pterygoid process, respectively, into the optic canal, the foramen rotundum, and the pterygoid canal. At the same time, in the investigated group of children, we found a more frequent occurrence of bilateral pneumatization of the lesser wings, greater wings, and pterygoid processes than unilateral pneumatization. In the case of rare subtypes of sphenoid sinus extensions into the sphenoid bone processes, unilateral occurrence of a given subtype, in the right or left sphenoid sinus, was observed more frequently.

## Figures and Tables

**Table 1:** Types and subtypes of the sphenoid sinus extensions into the sphenoid bone processes in the frontal plane.

Type	Subtypes	The Sphenoid Sinus Pneumatization into the Sphenoid Bone Processes
I		the sphenoid bone’s lesser wing
	Ia	above the optic canal
	Ib	below the optic canal
	Ic	simultaneously above and below the optic canal
II		the sphenoid bone’s greater wing
	IIa	above the foramen rotundum
	IIb	below the foramen rotundum
	IIc	simultaneously above and below the foramen rotundum
III		the sphenoid bone’s pterygoid process
	IIIa	laterally to the pterygoid canal
	IIIb	medially to the pterygoid canal
	IIIc	simultaneously laterally and medially to the pterygoid canal

**Table 2:** Frequency of occurrence of types and subtypes of right and left sphenoid sinus extensions into the sphenoid bone processes in the study group (N = 432).

Type	Subtypes	Sphenoid Sinus				*p*
		Right	In Both	Left	None	
		n (%)	n (%)	n (%)	n (%)	
I		22 (5.09)	33 (7.64)	29 (6.71)	348 (80.56)	<0.0001
	Ia	2 (0.46)	1 (0.23)	3 (0.69)	426 (98.61)	<0.0001
	Ib	20 (4.63)	28 (6.48)	27 (6.25)	357 (82.64)	<0.0001
	Ic	3 (0.69)	1 (0.23)	2 (0.46)	426 (98.61)	<0.0001
II		29 (6.71)	93 (21.53)	34 (7.87)	276 (63.89)	<0.0001
	IIa	1 (0.23)	1 (0.23)	2 (0.46)	428 (99.07)	<0.0001
	IIb	28 (6.48)	87 (20.14)	33 (7.64)	284 (65.74)	<0.0001
	IIc	3 (0.69)	2 (0.46)	2 (0.46)	425 (98.38)	<0.0001
III		23 (5.32)	60 (13.89)	25 (5.79)	324 (75.00)	<0.0001
	IIIa	25 (5.79)	28 (6.48)	28 (6.48)	351 (81.25)	<0.0001
	IIIb	4 (0.93)	1 (0.23)	5 (1.16)	422 (97.69)	<0.0001
	IIIc	12 (2.78)	13 (3.01)	10 (2.31)	397 (91.90)	<0.0001

**Table 3:** Frequency of occurrence of types and subtypes of right and left sphenoid sinus extensions into the sphenoid bone processes in girls and boys, respectively.

Type	Subtypes	Right Sphenoid Sinus			Left Sphenoid Sinus		
		Girls (N = 216)	Boys (N = 216)	*p*	Girls (N = 216)	Boys (N = 216)	*p*
		n (%)	n (%)		n (%)	n (%)	
I		30 (13.89)	25 (11.57)	0.5640	35 (16.20)	27 (12.50)	0.3368
	Ia	0 (0.0)	3 (1.39)	0.2483	1 (0.46)	3 (1.39)	0.6233
	Ib	28 (12.96)	20 (9.26)	0.2838	33 (15.28)	22 (10.19)	0.1484
	Ic	2 (0.93)	2 (0.93)	1.0	1 (0.46)	2 (0.93)	1.0
II		74 (34.26)	48 (22.22)	**0.0074**	69 (31.94)	58 (26.85)	0.2909
	IIa	1 (0.46)	1 (0.46)	1.0	2 (0.93)	1 (0.46)	1.0
	IIb	70 (32.41)	45 (20.83)	**0.0088**	64 (29.63)	56 (25.93)	0.4522
	IIc	3 (1.39)	2 (0.93)	1.0	3 (1.39)	1 (0.46)	0.6233
III		47 (21.76)	36 (16.67)	0.2219	42 (19.44)	43 (19.91)	1.0
	IIIa	31 (14.35)	22 (10.19)	0.2405	28 (12.96)	28 (12.96)	1.0
	IIIb	3 (1.39)	2 (0.93)	1.0	1 (0.46)	5 (2.31)	0.2155
	IIIc	13 (6.02)	12 (5.56)	1.0	13 (6.02)	10 (4.63)	0.6691

*p*—girls vs. boys.

## Data Availability

The data that support the findings of this study are available from the corresponding author: Przemysław Kiciński, e-mail: przemyslaw.kicinski@umed.lodz.pl or kicinskiprzemko@gmail.com.
